# Bourbon Virus in Field-Collected Ticks, Missouri, USA

**DOI:** 10.3201/eid2312.170532

**Published:** 2017-12

**Authors:** Harry M. Savage, Kristen L. Burkhalter, Marvin S. Godsey, Nicholas A. Panella, David C. Ashley, William L. Nicholson, Amy J. Lambert

**Affiliations:** Centers for Disease Control and Prevention, Fort Collins, Colorado, USA (H.M. Savage, K.L. Burkhalter, M.S. Godsey, Jr., N.A. Panella, A.J. Lambert);; Missouri Western State University, St. Joseph, Missouri, USA (D.C. Ashley);; Centers for Disease Control and Prevention, Atlanta, Georgia, USA (W.L. Nicholson)

**Keywords:** Bourbon virus, Amblyomma americanum, Thogotovirus, ticks, vector-borne infections, Kansas, Missouri, viruses, arboviruses, Oklahoma, United States

## Abstract

Bourbon virus (BRBV) was first isolated in 2014 from a resident of Bourbon County, Kansas, USA, who died of the infection. In 2015, an ill Payne County, Oklahoma, resident tested positive for antibodies to BRBV, before fully recovering. We retrospectively tested for BRBV in 39,096 ticks from northwestern Missouri, located 240 km from Bourbon County, Kansas. We detected BRBV in 3 pools of *Amblyomma americanum* (L*.*) ticks: 1 pool of male adults and 2 pools of nymphs. Detection of BRBV in *A. americanum,* a species that is aggressive, feeds on humans, and is abundant in Kansas and Oklahoma, supports the premise that *A. americanum* is a vector of BRBV to humans. BRBV has not been detected in nonhuman vertebrates, and its natural history remains largely unknown.

Bourbon virus (BRBV) was first isolated from blood samples from a hospitalized male resident of Bourbon County, Kansas, USA (*1*). He was >50 years of age and previously healthy. Several days before becoming ill in late spring 2014, he reported several tick bites and an engorged tick on his shoulder. Initial symptoms included nausea, weakness, and diarrhea (*1*). On day 2 after symptom onset, he had experienced fever, anorexia, chills, headache, myalgia, and arthralgia. On day 4 after onset, he was hospitalized. Physical examination found a papular rash on his trunk. The patient had a temperature of 37.3°C and laboratory findings of leukopenia, lymphopenia, thrombocytopenia, hyponatremia, and increased levels of aspartate aminotransferase and alanine aminotransferase. He was treated with intravenous fluids and doxycycline for possible tickborne illness. Serologic assays for the causative agents of Rocky Mountain spotted fever, tularemia, brucellosis, babesiosis, and Q fever were negative, as were molecular tests for *Ehrlichia* spp. and *Anaplasma phagocytophilum* and blood smears for *Babesia* (*1*). The patient died 11 days after symptom onset.

Virologic tests on EDTA-treated blood and separated serum collected from the patient on day 9 after symptom onset were negative for Heartland virus (HRTV; family *Bunyaviridae*, genus *Phlebovirus*) (*1*), a recently described tickborne virus (*2*,*3*). However, during plaque reduction neutralization tests for HRTV antibody, heterologous (non-HRTV) viral plaques were observed. Subsequently, plaque assay results revealed distinct plaques 3 days after inoculation within wells inoculated with blood and serum (*1*). Electron microscopy of virus particles demonstrated filamentous and spherical particles consistent with the morphology of the family *Orthomyxoviridae*. Full-length sequencing and phylogenic analysis demonstrated that the virus was new, most closely but distantly related to the Old World virus Dhori virus, and a member of the genus *Thogotovirus* (*1**,**4*). This new virus was named Bourbon virus after the county of residence of the patient. BRBV is the first human pathogen of the genus *Thogotovirus* to be identified in the New World (*4*).

In May 2015, the Centers for Disease Control and Prevention (CDC) and the Oklahoma State Department of Health reported that a Payne County, Oklahoma, USA, resident became ill and tested positive for antibodies to BRBV by plaque reduction neutralization tests (E. Staples, O. Kosoy, CDC, pers. comm., 2016 Dec 5). The patient recovered fully.

In response to the report of the fatal BRBV case from eastern Kansas in 2014, we retrospectively tested ticks for BRBV that were collected during spring and summer 2013 from 6 sites in northwestern Missouri, ≈240 km from Bourbon County (Figure 1). We had originally collected, identified, pooled, and processed these tick samples as part of an ongoing HRTV surveillance program (*5*). The goals of our retrospective analysis were to determine whether BRBV was present in the neighboring state of Missouri, to incriminate possible vector species, and to determine which life history stages are involved in virus transmission to humans.

**Figure 1:**
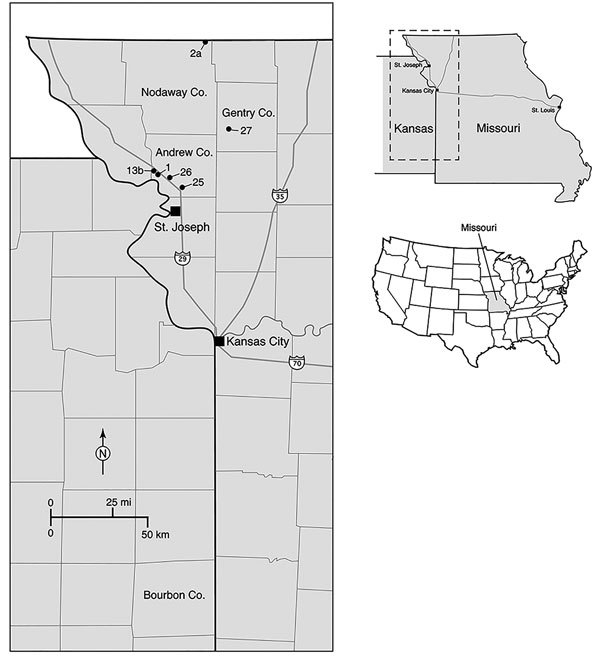
Locations of 6 tick sampling sites surveyed in northwestern Missouri, USA, during 2013 (indicated by site numbers), showing proximity of site to Bourbon County, Kansas (bottom center of map). Inset maps show location of area in main map (top, dashed box) and location of state of Missouri in the United States (bottom, gray shading). Co., County.

## Materials and Methods

### Tick Collections

We collected ticks at 6 sites in 3 counties of northwestern Missouri: Andrew, Gentry, and Nodaway (Figure 1). Five sites were properties owned by HRTV patients (sites 1, 2a, and 25–27), and 1 site (13b) was state recreational land, Honey Creek Conservation Area (*5*). Ticks were collected during three 1-week-long field trips in 2013; tick collections occurred on April 22–25, June 10–13, and July 22–25. We collected ticks primarily by flagging and secondarily by use of carbon dioxide–baited tick traps as described previously (*3**,**5*). We then froze the ticks on dry ice and shipped them to CDC (Fort Collins, CO, USA) for processing and testing.

### Tick Processing, RNA Extraction, and Virus Detection

We identified ticks to species, sex, and life history stage using microscopes on refrigerated tables and standard taxonomic references (*5*) and grouped them into pools by site, collection date, collection type, species, sex, and stage. We homogenized tick pools in 1 mL chilled bovine albumin-1 (BA-1) in glass TenBroeck grinders (Fischer Scientific, Pittsburgh, PA, USA) as described previously (*3**,**5*). After centrifugation, we removed a 125-μL aliquot of supernatant from each tick pool homogenate and placed the aliquot into an identically numbered tube for RNA extraction. The remaining homogenates were held at −80°C for future confirmatory testing.

We extracted RNA from a 100-μL sample removed from each aliquot tube using the QIAmp Virus BioRobot 9604 kit on a BioRobot Universal platform (both from QIAGEN, Valencia CA, USA) according to the manufacturer’s protocol. Sequence and reporter information for BRBV primer/probe sets nucleoprotein (NP) 1 and polymerase basic (PB) 1, which we used for virus detection and confirmation, respectively, are as previously described (*4*). We screened all samples for BRBV by using primer/probe set NP1 in a real-time reverse transcription PCR (rRT-PCR) as described for HRTV (*5*). Pools positive in the screening assay were confirmed by reextracting RNA from the original tick homogenate and performing the quantitative rRT-PCR with 2 primer/probe sets, primer/probe sets NP1 and PB1. We considered pools BRBV positive if crossing threshold scores for both primer/probe sets were <37.

To estimate the detection limit associated with a crossing threshold of 37, we spiked tick pools of specimens from an *A. americanum* colony with serial dilutions of BRBV, original strain (*1*,*4*). Pools comprised either 5 adult females or 25 nymphs and were ground in 1 mL BA-1. We tested 14 pools of adults and 14 pools of nymphs with each primer set.

### Plaque Assays to Detect Viable Virus

We tested tick homogenates from rRT-PCR–positive samples for viable virus with 2-step cell culture assay using human hepatoma cell line 7 (HuH-7) following a published protocol (*5*). Initially, we inoculated a 200-μL sample of tick homogenate into HuH-7 cells in separate T-25 flasks, monitored the flasks daily for cytopathic effect, and harvested on day 5 or 6. We then used this first viral harvest (V1) to inoculate HuH-7 cells in 6-well plates and counted plaques to estimate titer (*5*).

### High-Throughput Sequencing and Phylogenetic Analysis

We extracted and purified BRBV RNA from HuH-7 V1 harvest from 2 pools that were rRT-PCR positive as previously described (*1**,**4*). We then conducted high-throughput sequencing on an Ion Torrent PGM sequencer (Life Technologies, Grand Island, NY, USA) and analyzed sequence data from repeated runs using the CLC Genomics Workbench 7.5.1 (CLCbio, Cambridge, MA, USA) and NGen 4 (DNAstar, Madison, WI, USA) software program as previously described (*4*). The total approximated average genome coverage across all genomic segments was 1,000×. We determined open reading frames using the EditSeq function of the Lasergene 9 package (DNAstar) and conducted phylogenetic analysis on nucleotide and amino acid sequences using MEGA5 software (*6*).

## Results

### Detection of BRBV in Ticks and Infection Rates

Based on spiked tick pools comprising 5 *A. americanum* adult females or 25 nymphs, ground in 1 mL BA-1, the cutoff crossing threshold of 37 corresponded to a detection limit of 10^2.6^ PFU/mL or pool (95% CI 10^2.5^–10^2.7^) for primer set NP1. The crossing threshold of 37 corresponded to a detection limit of 10^1.4^ PFU per mL or pool (95% CI 10^1.3^–10^1.5^) for primer set PB1. Results from adult and nymphal pools were not statistically different.

We tested 39,096 ticks representing 5 species collected from 6 sites in northwestern Missouri (Figure 1; Table 1). However, 2 species, *A. americanum* (L.) (97.6%) and *Dermacentor variabilis* (Say) (2.3%), accounted for 99.9% of ticks collected.

**Table 1:** Characteristics of a retrospective study of Bourbon virus in field-collected ticks, Missouri, USA, 2013

Site	*Amblyomma americanum*				*Dermacentor variabilis*			*Haemaphysalis leporispalustris* nymphs	*Ixodes dentatus*		Total
	Adults	Nymphs	Larvae		Adults	Nymphs	Larvae		Adults	Nymphs	
1	2,473	7,534	100		162	27			2	8	10,306
2a	267	2,822			141	14	28	1	1	4	3,278
13b	2,811	8,847	944		396	6	2	1	3	9	13,020*
25					6						6
26	389	4,528	696		91	4					5,708
27	252	6,478	11		36					1	6,778
Total	6,192	30,209	1,751		832	51	30	2	6	22	39,096*

*Includes 1 *I. scapularis* nymph collected at site 13b on June 11, 2013.

We tested an aliquot from all 3,073 tick pools from Missouri collections from 2013 by rRT-PCR using the screening primer/probe set NP1. Three pools were positive in the screening assay. Reextraction and testing of the original tick homogenates using both primer/probe sets NP1 and PB1 confirmed BRBV RNA in all 3 pools. All 3 tick pools yielded viable virus in cell culture. All 3 positive pools comprised *A. americanum* ticks (Table 2). One pool comprised 4 male adult ticks collected at site 2a on June 12; the other 2 pools each comprised 25 nymphs collected at site 27 on July 24.

**Table 2:** Bourbon virus confirmed by real-time reverse transcription PCR in pools of *Amblyomma americanum* ticks, Missouri, USA, 2013

Pool no.	County	Site	Collection date	Stage	Sex*	No. specimens
MO-2013-1246	Nodaway	2a	Jun 12	Adult	M	4
MO-2013-2499	Gentry	27	Jul 24	Nymph	–	25
MO-2013-2530	Gentry	27	Jul 24	Nymph	–	25

*–, nymphs cannot be sexed.

The maximum-likelihood estimate (*7*) of the infection prevalence per 1,000 ticks, for nymphs of *A. americanum* from site 27 on July 24, 2013, the only day that this site was sampled, was 0.31 (95% CI 0.06–1.01), or ≈1 infected nymph per 3,226 collected nymphs. The infection prevalence for *A. americanum* nymphs from all sites combined during the entire 2013 season was 0.07 (95% CI 0.01–0.22), or ≈1 infected nymph per 14,286 collected nymphs.

The infection prevalence for adult male *A. americanum* ticks from site 2a on June 12, 2013, was 19.11 (95% CI 1.13–90.06),; for adult male *A. americanum* ticks from site 2a during the entire 2013 season was it .35 (95% CI 0.42–35.19); and for all adult male *A. americanum* ticks from all sites combined during the 2013 season it was 0.32 (95% CI 0.02–1.53). The 95% CI for difference of proportions (*7*) between the infection prevalence for male adults and nymphs from all sites combined during the 2013 season includes zero (95% CI −1.46 to 0.13), indicating that infection prevalence for male adults and nymphs did not significantly differ.

### Phylogenetic Analyses

**Figure 2:**
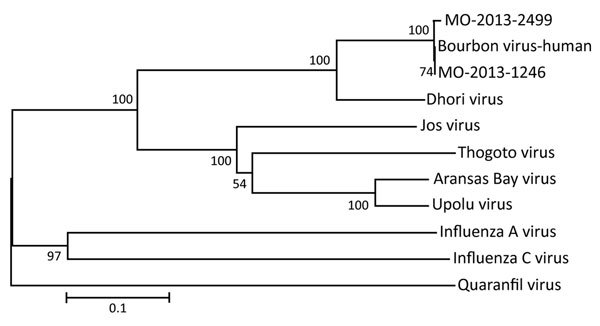
Phylogenetic analyses of partial basic 2 sequences of selected orthomyxoviruses. Bourbon virus sequences from 2 pools of *Amblyomma americanum* ticks (male adults, MO-2013-1246; nymphs, MO-2013-2499) collected in Missouri, USA, during 2013 grouped with the sequence of the original Bourbon virus isolated from a man who died in Bourbon County, Kansas, USA, during 2014. The evolutionary history was inferred using the neighbor-joining method with 2,000 replicates for bootstrap testing. The tree is drawn to scale, with branch lengths in the same units as those of the evolutionary distances used to infer the phylogenetic tree. The evolutionary distances were computed using the Poisson correction method. Scale bar indicates number of amino acid substitutions per site.

To confirm the molecular identification of BRBV, we selected 2 pools, MO-2013-1246 of male adults and MO-2013-2499 of nymphs, for high-throughput sequencing and phylogenetic analysis. We deposited partial genomic sequence data in GenBank (accession nos. KY825740–KY825741). Analyses revealed 6 RNA segments for strain MO-2013-1246, as previously reported for the BRBV human strain (*4*). We conducted phylogenetic analysis on a 152-aa sequence of PB2 subunit of the polymerase protein (Figure 2) to assess relationships with the BRBV strain from the fatal human case and other selected members of the *Orthomyxoviridae*. The 3 BRBV strains form a lineage with 100% bootstrap support. The BRBV lineage is a sister group to, and mostly closely related to, Dhori virus. The BRBV-Dhori lineage appears as a sister group to a lineage of 4 tick-associated viruses and distantly related to the influenza viruses and Quaranfil virus.

The human BRBV strain from Kansas and tick pool MO-2013-1246 comprising male adult *A. ambylomma* ticks were very similar for the PB2 gene segment analyzed, sharing >99.0% sequence at the amino acid level and 95.0% identity at the RNA sequence level. Furthermore, the human BRBV strain from Kansas and tick pool MO-2013-2499 (nymphs) were very similar for the PB2 gene segment analyzed, sharing 99.0% sequence identity at the amino acid and RNA sequence levels.

## Discussion

We isolated BRBV from 3 pools of *A. americanum* ticks collected in northwestern Missouri: 1 pool of male adults and 2 pools of nymphs. The first detection of BRBV was from a fatal case in a man from nearby Bourbon County, Kansas (*1*); this man reported tick exposure and an engorged tick on his shoulder shortly before he became ill. Tick exposure combined with laboratory findings of leukopenia and thrombocytopenia suggested that BRBV might be transmitted to humans by ticks. Detection of BRBV in field-collected *A. americanum* ticks from Missouri supports the premise that *A. americanum*, a species that is aggressive, feeds on humans (*8*), and is abundant in Kansas and Oklahoma (*9*), the states where the 2 persons with BRBV infection resided, is a vector of BRBV to humans.

Tick transmission of BRBV also is consistent with our knowledge of the vector status and phylogenetic relationships within the genus *Thogotovirus* and related viruses in the family *Orthomyxoviridae* (Figure 2). Viruses that have been placed in the genus *Thogotovirus* include BRBV (*1*), Thogoto virus (*10**,**11*), Araguari virus (*10**,**12*), Dhori virus (*10**,**11*), Jos virus (*10**,**13*), and Upolu virus (*13**,**14*). To our knowledge, the closely related Aransas Bay virus (*14*) has not been placed in genus *Thogotovirus*. All of these viruses, except Araguari virus, which has been isolated only from vertebrates, are believed to be transmitted by a variety of hard and soft tick species (*15**–**22*). Of these tick-transmitted viruses (Figure 2), only Thogoto virus, Dhori virus, and BRBV have been associated with human disease (*1**,**18*), and only BRBV and Aransas Bay virus are known to occur in North America. Aransas Bay virus has been isolated from the soft tick *Ornithodoros capensis*, a parasite of seabirds (*19**,**23*).

Dhori virus, the virus most closely related to BRBV (*4*), is an Old World virus known from Europe, North Africa, and western and central Asia. Dhori virus has been isolated primarily from metastriate ticks (hard ticks other than genus *Ixodes*), including *Hyalomma dromedarii*, *H.* *marginatum* (reported as *H. plumbeum plumbeum* in the former Soviet Union), *H. scupense*, and *Dermacentor marginatus* (*16**,**18**,**20**,**24*). On rare occasions, Dhori virus also has been isolated from mosquitoes, including *Anopheles hyrcanus* (*24*), and from 1 mixed pool of *Aedes caspius caspius* and *Culex hortensis* mosquitoes collected near the Naryn River in Kyrgyzstan (*20*). Human disease associated with Dhori virus infection is characterized by acute illness with severe fever, headache, general weakness, and retrobulbar pain; encephalitis occurs in ≈40% of patients, and convalescence is long (2 months) (*18*). In addition, 5 laboratory infections resulting from aerosol exposure, 2 of which were characterized by encephalitis, have been reported (*25*).

Infection prevalence for BRBV in field-collected *A. americanum* ticks varied. The infection prevalence for nymphs, the stage with the largest sample size, was 0.31/1,000 or 1/3,226 nymphs at site 27. Nymphs collected at other sites were virus negative, resulting in a very low infection prevalence of 0.07/1,000 when all nymphal collections were combined for the 2013 season. For comparison, HRTV was detected in nymphs collected at 4 of the 6 sampled sites, and the infection prevalence for HRTV in nymphs from all sites during the 2013 season was 1.79/1,000, or 1 infected nymph of 559 nymphs tested (*5*).

The BRBV infection prevalence for adult male *A. americanun* ticks from site 2a on June 12, 2013, was very high (19.11/1,000), whereas the infection prevalence for adult male *A. americanum* ticks from site 2a during the entire 2013 season was 7.35/1,000, or 1 infected male adult among every 136 tested. However, the infection prevalence for all adult male *A. americanum* ticks from all sites combined during the 2013 season was 0.32/1,000, or 1 of 3,125 adult male ticks tested. Infection prevalence for adult male ticks appears higher than for nymphs; however, BRBV infection prevalence for male adults and nymphs from all sites combined during the 2013 season did not differ significantly.

Infection prevalence for BRBV in potential vectors remains poorly known; our interpretations are preliminary and await additional field studies. However, the very low infection prevalence for nymphs and varying rates for male adults suggest the possibility that other transmission cycles for BRBV might exist and that *A. americanum* ticks, although most likely an important vector to humans because of their aggressive host-seeking behavior and preference for medium and large mammals, might not be an important enzootic vector of BRBV. We hypothesize that *A. americanum* ticks acquire BRBV from occasional blood meals from >1 vertebrate hosts; that the virus successfully replicates and is transstadially transmitted in *A. americanum* ticks; and that *A. americanum* ticks transmit the virus to incidental hosts, such as humans. The pool of male adult *A. americanum* ticks, MO-2013-1246, also was positive for HRTV (*5*), suggesting some overlap in the transmission cycles of HRTV and BRBV. BRBV and antibodies to BRBV have not been detected in vertebrates, other than the 2 humans, and the natural history of the virus remains unknown.
